# Multi-Stream Fusion Network for Skeleton-Based Construction Worker Action Recognition

**DOI:** 10.3390/s23239350

**Published:** 2023-11-23

**Authors:** Yuanyuan Tian, Yan Liang, Haibin Yang, Jiayu Chen

**Affiliations:** 1 Department of Architecture and Civil Engineering, City University of Hong Kong, Hong Kong 999077, China; yuanytian2-c@my.cityu.edu.hk; 2 Key Laboratory for Resilient Infrastructures of Coastal Cities (MOE), College of Civil and Transportation Engineering, Shenzhen University, Shenzhen 518060, China; liangyan2021@email.szu.edu.cn (Y.L.); haibin_yang@szu.edu.cn (H.Y.); 3 School of Civil Engineering, Tsinghua University, Beijing 100084, China

**Keywords:** construction worker action recognition, deep learning algorithm, 3D skeleton data, multi-stream network

## Abstract

The global concern regarding the monitoring of construction workers’ activities necessitates an efficient means of continuous monitoring for timely action recognition at construction sites. This paper introduces a novel approach—the multi-scale graph strategy—to enhance feature extraction in complex networks. At the core of this strategy lies the multi-feature fusion network (MF-Net), which employs multiple scale graphs in distinct network streams to capture both local and global features of crucial joints. This approach extends beyond local relationships to encompass broader connections, including those between the head and foot, as well as interactions like those involving the head and neck. By integrating diverse scale graphs into distinct network streams, we effectively incorporate physically unrelated information, aiding in the extraction of vital local joint contour features. Furthermore, we introduce velocity and acceleration as temporal features, fusing them with spatial features to enhance informational efficacy and the model’s performance. Finally, efficiency-enhancing measures, such as a bottleneck structure and a branch-wise attention block, are implemented to optimize computational resources while enhancing feature discriminability. The significance of this paper lies in improving the management model of the construction industry, ultimately aiming to enhance the health and work efficiency of workers.

## 1. Introduction

In the construction industry, construction workers constitute the fundamental unit, and their behavior significantly contributes to workplace accidents and injuries. Managing and controlling workers, who are inherently dynamic, prove to be a major challenge on construction sites. A concerning 80–90% of accidents are closely tied to unsafe actions and behaviors exhibited by workers [1]. Traditional approaches to measure and improve worker behavior, such as self-reports, observations, and direct measurements, are known but are time- and labor-intensive.

Safety risks associated with worker posture are a significant concern in construction projects. Construction workers typically face the risk of immediate or long-term injuries, including unsafe and awkward activities [2] and falls from ladders. These injuries arise due to repetitive movements, high force exertion, vibrations, and awkward body postures, all of which are common occurrences among construction workers [3]. Various efforts, including career training, education, and site manager observations, have been implemented to mitigate posture-related safety risks. Nevertheless, current methods still lack effectiveness in managing these risks.

Recently, there has been a growing body of research dedicated to the automation of information extraction from skeleton data using deep learning algorithms. Recurrent Neural Networks (RNNs) [4] have emerged as a prominent choice for skeleton-based action recognition due to their effectiveness in handling sequential data. On the other hand, Convolutional Neural Networks (CNNs) [5] are often employed to transform skeleton data into image-like formats. RNN-based methods are highly effective in handling skeleton sequences, being naturally suited for modeling time series data and capturing temporal dependencies. To further enhance the understanding of the temporal context within skeleton sequences, other RNN-based techniques, like Long Short-Term Memory (LSTM) [6] and Gated Recurrent Units (GRUs) [7], have been incorporated into skeleton-based action recognition methodologies. The integration of CNNs alongside RNNs offers a complementary approach, as CNN architectures are proficient in capturing spatial cues present in input data, addressing a potential limitation of RNN-based methods.

However, these two approaches lack the ability to comprehensively capture the intricate dependencies among correlated joints of the human body. A more robust analysis algorithm, the Graph Convolutional Network (GCN) [8,9,10], was introduced to enhance skeleton-based action recognition. This was inspired by the understanding that human 3D skeleton data inherently forms a topological graph distinct from the sequence vector or pseudo-image treatment seen in RNN-based or CNN-based methods. In recent times, the GCN has gained prominence in this task due to its effective representation of graph-structured data. There are generally two types of prevalent graph-related neural networks: graph and GCN [8]; our primary focus in this survey is on the latter. Over the past two years, several classic methods have been proposed in this domain. For instance, Yan et al. [11] introduced a method in which skeleton sequences are embedded into multiple graphs. In this approach, the joints within a frame of the sequence act as nodes in the graph, with the connections between these joints representing the spatial edges of the graph.

Therefore, the majority of these approaches have traditionally focused on a single, encompassing graph based on the natural connections within the human body structure. This approach confines a node to capturing only a specific type of spatial structure, resulting in an inability of existing GCN methods to fully learn features within a single fixed graph, which typically represents the physical relations of nodes. The convolution operator, which is integral to GCN, heavily relies on meaningful graph structures to capture extensive local and contour features in spatial data. Thus, the significance of developing meaningful graph structures that promise to capture more local features and contour features in spatial data cannot be overstated. Future research in this domain should concentrate on refining GCN methodologies to achieve more robust feature learning within dynamic graph structures, ultimately advancing the field of skeleton-based action recognition.

In this paper, our goal is to introduce a multi-scale graph strategy designed to acquire not only local features, such as the relationship between the head and neck, but also to capture broader connections, like the relationship between the head and foot. We utilize varying scale graphs in distinct network streams, strategically incorporating physically unrelated information. This addition aids in enabling the network to capture local features of individual joints and crucial contour features of significant joints. Moreover, we incorporate velocity and acceleration as novel temporal features, which are fused with spatial features, enhancing the depth of information. The primary contributions of this study can be summarized as follows:

1. We devised the multi-feature fusion network (MF-Net) incorporating three-level spatial features (body-level, part-level, joint-level) as different network streams. This multi-stream strategic addition facilitates the network in capturing both significant local and global features of workers’ joints.
2. Going beyond diverse spatial features, we innovatively introduced the velocity and acceleration of each joint as temporal features. Meanwhile, we proposed a spatial–temporal two-step fusion strategy, effectively correlating high-level feature maps from multiple streams. These features were seamlessly integrated with three-scale spatial features, resulting in robust and efficient improvement in the performance of action recognition accuracy. We struck a balance between the independent learning of feature streams and ensuring adequate correlation of fusion streams to guarantee fusion performance.
3. In order to enhance the efficiency of our model, we implemented a bottleneck structure that effectively reduces computational costs during parameter tuning and model inference. This structure is further complemented by the utilization of two temporal kernel sizes, allowing us to cover different receptive fields. Additionally, we introduced a branch-wise attention block, which employs three attention blocks to calculate the attention weights for different streams, contributing to the further refinement of our model’s efficiency.

## 2. Literature Review

### 2.1. Action Recognition Based on Skeleton Data

Action recognition holds significant importance and poses a notable challenge across various industries. Traditional approaches in construction project information analysis primarily rely on self-reporting and observations by professionals [12]. These approaches encompass vision-based [13,14,15] and wearable sensor-based methods. For instance, Roberts et al. [16] extensively utilized 317 annotated videos to analyze the operations of bricklaying and plastering workers. Beyond conventional cameras, the utilization of RGB-D cameras in researching construction worker operations has garnered considerable attention. Khosrowpour et al. [15] introduced a supervised machine approach employing RGB-D sensors to predict worker activities, achieving an average accuracy of 76% in worker activity recognition.

Furthermore, Li et al. [17] introduced a novel approach based on geometric algebra to represent shape and motion, emphasizing the importance of considering both joints and bones in skeleton data. This representation effectively harnesses the information contained in skeleton sequences. In a similar vein, Liu et al. [18] utilized enhanced skeleton visualization techniques to represent skeleton data, enhancing the visual aspects of the information. Additionally, Carlos et al. [19] proposed a unique representation known as SkeleMotion, which focuses on encoding motion information. SkeleMotion achieves this by explicitly computing the magnitude and orientation values of skeleton joints, providing a valuable perspective on the temporal dynamics of skeletal data.

### 2.2. Deep Learning for Action Recognition

Conventional methods for skeleton-based action recognition primarily center around Machine Learning (ML), relying heavily on handcrafted features to represent the human body’s skeleton [20,21,22]. However, this approach to data processing is inherently intricate and time-consuming, making it better suited for small- or medium-sized datasets. In the realm of evolving technologies, particularly the advancement of Deep Neural Networks (DNNs), data-driven approaches, including RNNs, CNNs, and GCNs, have gained significant traction and popularity.

#### 2.2.1. RNN-Based Approaches

The recursive connection within the RNN structure is established by utilizing the output from the previous time step as the input for the current time step [23]. This mechanism has demonstrated its effectiveness in processing sequential data. In a similar vein, LSTM extends the capabilities of the standard RNN by introducing gates and linear memory units, addressing issues such as gradient problems and long-term temporal modeling.

Considering the aspect of spatial–temporal modeling, which is a fundamental principle in action recognition tasks, it becomes evident that the spatial modeling ability of RNN-based architectures is a limiting factor. This limitation often hampers the performance of related methods, failing to achieve competitive results [24]. Recently, Hong and Liang [25] proposed an innovative two-stream RNN architecture, effectively modeling both temporal dynamics and spatial configurations for skeleton data. This involved an exchange of the skeleton that was applied as a preprocessing step to enhance spatial domain learning. Song. et al. [26] introduced a spatial attention module that adaptively allocates varying levels of attention to different joints within each frame of the input skeleton. Additionally, a temporal attention module allocates distinct attention to different frames. In a relevant domain, Antwi-Afari et al. [27] evaluated three types of RNN-based networks for the automated recognition and classification of construction workers’ awkward postures, achieving the highest performance with a GRU model, at approximately 99.01%.

#### 2.2.2. CNN-Based Approaches

CNNs have seen application in skeleton-based action recognition. Unlike RNNs, CNN models demonstrate a remarkable ability to efficiently learn high-level semantic cues due to their natural capability to extract higher-level information. However, CNNs are primarily geared towards image-based tasks, and adapting them to action recognition based on skeleton sequences poses a significant challenge, given the inherently time-dependent nature of the task. Striking a balance and fully leveraging spatial and temporal information in a CNN-based architecture remains an ongoing challenge.

The concept of a temporal convolutional network (TCN) was initially introduced by Lea et al. [28] for human action segmentation. They employed an encoder–decoder TCN approach throughout a video, and the outcomes demonstrated that TCN architectures could achieve comparable functions to LSTM while offering faster training times. Building upon this, Jia et al. [29] introduced a two-stream TCN architecture for skeleton-based human action recognition. This approach comprised an intra-frame stream and an inter-frame stream. For intra-frame features, the human body was divided into five relevant joint subgroups, with each part’s features concatenated to represent the spatial domain. Regarding the inter-frame stream, they utilized the coordinate difference between adjacent frames as the representation. The entire model was constructed with twelve basic residual blocks, each containing two TCN layers followed by activation functions and dropout layers.

#### 2.2.3. GCN-Based Approaches

Recent research places more attention on graph data, such as e-commence recommended systems [30], chemistry molecules [31], social networks [32], citation networks [33], and so on. GCN-based methods have garnered increasing interest in skeleton-based action recognition due to their expertise in handling skeleton data within a non-Euclidean space [34,35,36]. The construction of the GCN on the graph primarily follows a spatial perspective [37], involving the direct application of convolutional filters to the graph nodes.

In the realm of skeleton-based action recognition, various classic methods operate directly on graph-structured data, allowing the propagation of local messages [38,39]. To illustrate this, consider the strong interdependence between the two hands during a clapping action, a connection not inherently present in the graph based on the human body’s structural connections. For instance, Yan et al. [11] introduced an influential model called Spatial Temporal Graph Convolutional Networks (ST-GCNs) for skeleton-based action recognition. This network constructs a spatial–temporal graph with joints as the graph vertices, incorporating the natural connectivity of both human body structures and the temporal dimension as graph edges. Subsequently, higher-level feature maps on the graph, derived from ST-GCNs, are classified into corresponding action categories using a standard SoftMax classifier. Building on this foundation, there has been a growing emphasis on leveraging Graph Convolutional Networks (GCNs) for skeleton-based action recognition, leading to several related studies. Li et al. [36] introduced the Action-Structural GCN (AS-GCN), which not only recognizes a person’s action but also employs a multitask learning strategy to predict the subject’s potential next pose. The graph created in this work captures richer dependencies among joints through two key modules: actional links and structural links. This progress opens up new avenues for enhancing the understanding of complex actions in skeleton-based action recognition.

## 3. Methodology

The content of this section can be summarized as follows: Firstly, we introduce MF-Net, a robust and efficient multi-feature network designed for learning spatial–temporal feature sequences. Secondly, in terms of spatial features, we design a hierarchical skeleton topology (body, part, joint) and utilize a graph convolution network to extract these features. For temporal features, we incorporate velocity and acceleration as novel features fused with spatial features. Thirdly, TCN serves as the main network for recognizing sequence information. To optimize our model’s efficiency, we implement a bottleneck structure, effectively reducing computational costs during parameter tuning and model inference. Additionally, we set two different temporal kernel sizes to obtain varying receptive fields. Finally, we propose a two-stage fusion strategy integrating a branch-wise attention block to correlate high-level feature maps from multiple streams. We carefully balance the independent learning of feature streams and ensure adequate correlation of fusion streams to optimize fusion performance. Furthermore, we introduce attention mechanisms to compute attention weights for different branches, further enhancing the discriminative capability of the features.

### 3.1. Pipeline of Multi-Scale Network

**Figure 1:**
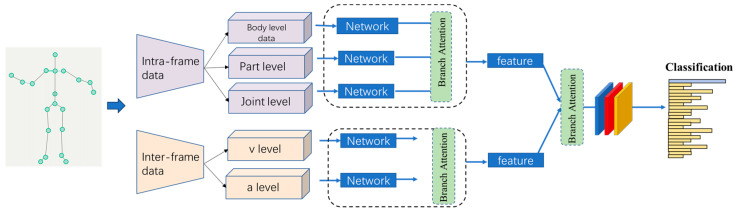
The architecture of the MF-Net.

The network comprises two branches, each incorporating five sub-streams based on input features, as shown in Figure 1. Branch 1 represents the spatial features. Before entering the sequence network, we employ a GCN to extract spatial structural features from each frame. A simplistic fusion approach involves concatenating these features at the feature level and passing them through a fully connected layer. However, this approach is not optimal due to inconsistencies in features across different streams.

#### 3.1.1. GCN Model

In contrast to CNN models, graph neural networks (GNNs) [8] are designed to process graph-structured data. In essence, the input should depict a set of vertices (or nodes) and a structure that delineates the relationships between them [40]. Each node undergoes an update to a latent feature vector containing information about its neighborhood, resembling a convolution operation, and leading to the nomenclature of GCN. This latent node representation, often referred to as an embedding, can be of any chosen length. These embeddings can be employed for node prediction, or they can be aggregated to derive predictions at the graph level, providing information about the graph as a whole. Concurrently, various GNN variants have been utilized to address diverse tasks, such as graph classification [37], relational reasoning tasks [41], and node classification in extensive graphs [42].

**Figure 2:**
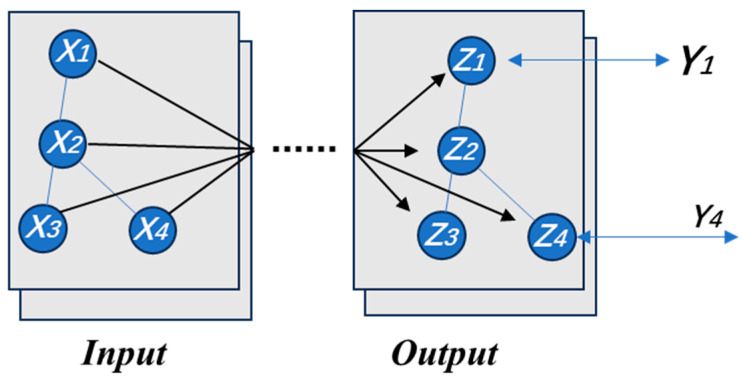
A graphical explanation of the GCN model.

Figure 2 presents a visual description of the GCN. In this example, at the input layer, there are four nodes, and each node takes an input vector of dimension C, together forming the initial activation $H_{0}$. After passing through several hidden layers, the activation is then converted into the values on the output layer nodes $Z_{i}$ of each dimension F. These are then compared with the partial labels $Y_{i}$ to generate a loss for the model to train on. Normally, a graph G(V,E) consists of vertices V and edges E, where each edge in E is a pair of two vertices. A walk is a sequence of nodes in a graph, wherein consecutive nodes are connected by an edge. Each graph can be represented by an adjacency matrix A of size n×n, where $A_{i,j}=1$ represents an edge between vertices $V_{i}$ and $V_{j}$, and $A_{i,j}=0$ indicates no connection between them. Vertices and edges attributes are features that possess a single value for each node and edge of a graph.

To perform the convolution operation in a GCN, the skeleton graph is transformed into the adjacency matrix A. When the skeletal joints $J_{i}$ and $J_{j}$ are connected in the skeleton graph, the value of $A_{ij}$ is set to 1; otherwise, it is set to 0. Therefore, different adjacency matrices A can represent various skeleton topological structures through the aggregation of information. Specifically, each skeleton frame can be converted into a graph G(V,E) to represent intra-body connections. V constitutes the joint set representing spatial features, while *E* is the set of edges between joints used to represent structural features. Based on the skeleton data X and its corresponding adjacency matrix A, the convolution operation in a GCN can be formulated as shown in Equation (1):

(1) $$f_{out}=\sigma \;\left(D^{-\frac{1}{2}}\tilde{A}D^{-\frac{1}{2}}f_{in}W\right)$$

where $\tilde{A}=A+I$ is the adjacency matrix of graph G with self-connection identity matrix I. D is the degree matrix of $\tilde{A}$. W is the learned weight matrix, and σ(·) denotes the activation function.

#### 3.1.2. TCN Sequence Model

**Figure 3:**
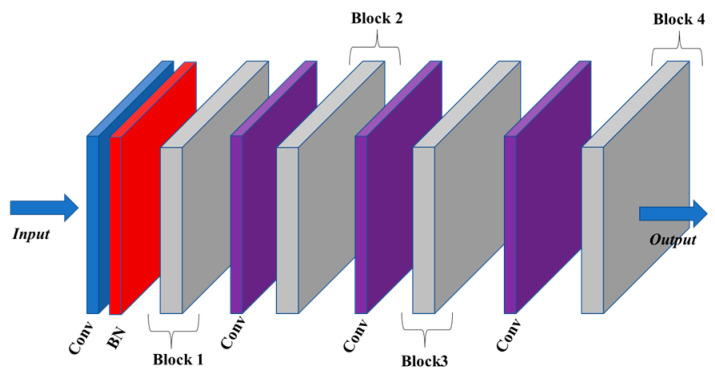
The overview of the proposed TCN architecture network.

Each stream comprises four blocks connected by a kernel size 1 temporal convolution layer, as shown in Figure 3. The output dimensions of blocks B1, B2, B3, and B4 are 32, 64, 128, and 256, respectively. These blocks are interconnected in a sequential manner. The input data are initially normalized by a Batch Normalization (BN) layer at the start of the network. Referring to the temporal convolution kernel sizes in related research, 3 is a popular choice [34]. Accordingly, the temporal convolution kernel sizes in our model are set to 3 and 5, resulting in corresponding receptive fields of 7 and 13, respectively. The TCN model is exclusively composed of convolutional structures and has exhibited excellent results in sequence modeling tasks without utilizing a recurrent structure. The temporal convolutional network can be regarded as a fusion of one-dimensional convolution and causal convolution:

(2) $$H^{\left(l+1\right)}=H^{\left(l\right)}W^{\left(l\right)}+Bias^{\left(l\right)}$$

where $H^{\left(l+1\right)}$ is the output feature of layer l, and $H^{\left(l\right)}\in {\mathbb{R}}^{input\_size}$ represents the input size. The $W^{\left(l\right)}$ and $B^{\left(l\right)}$ denote the learnable parameter matrices and the bias vectors, respectively.

A nuanced block structure known as the “bottleneck” was introduced [43]. Its operation involves the insertion of two convolution layers before and after a standard convolution layer. This strategic arrangement substantially reduces the overall network parameters. More precisely, it achieves this by decreasing the number of feature channels during convolution calculations with a reduction rate denoted as “r”. In this section, we replaced the conventional TCN block with a bottleneck structure to significantly expedite model training and responsiveness during implementation.

#### 3.1.3. Branch-Wise Attention

**Figure 4:**
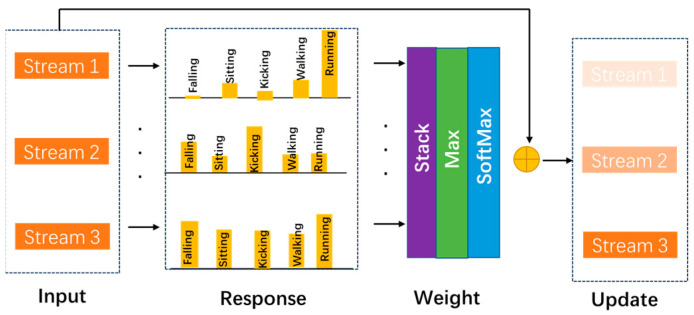
The overview of the proposed stream attention block.

In this work, we propose a two-step fusion strategy integrating an attention block method. Specifically, we focus on discovering the importance of different streams and branches. Inspired by split attention in the ResNeSt model [44], the attention block is designed as Figure 4. Taking a branch as an example, first, the features of different streams are taken as input. Secondly, they are passed through a fully connected layer (FC) with a Batch Norm layer and a ReLU function. Thirdly, the features of all streams are stacked. Subsequently, a max function is adopted to calculate the attention matrices, and a SoftMax function is utilized to determine the most vital stream. Finally, the features of all streams are concatenated as an integral skeleton representation with different attention weights. The stream attention block can be formulated as:

(3) $$f_{s}=f_{in}\left(s\right)⊙f_{max}\left(\theta \left(f_{in}\left(s\right)\right)W\right)+f_{in}\left(s\right)$$

(4) $$f_{out}=concat\left(\left\{f_{s}|s=1,2,3\right\}\right)$$

where $f_{in}\left(s\right)$ and $f_{s}$ denote input with and without the attention block, and θ(·) and $f_{max}$ represent ReLU activation and max function. And *W* is the learnable parameters of the FC layer.

### 3.2. Multi-Scale Input Features

In this research, we described the five different input streams. For the body-level input stream, we used the whole-body nodes based on human skeleton body to represent worker’s actions. For body-level and joint-level inputs, we split human body input five parts and abstract the relationship between important joints. Lastly, two-mode motion data were employed as inputs to encompass a broader range of motion features exhibited by workers.

#### 3.2.1. Part-Level Input

**Figure 5:**
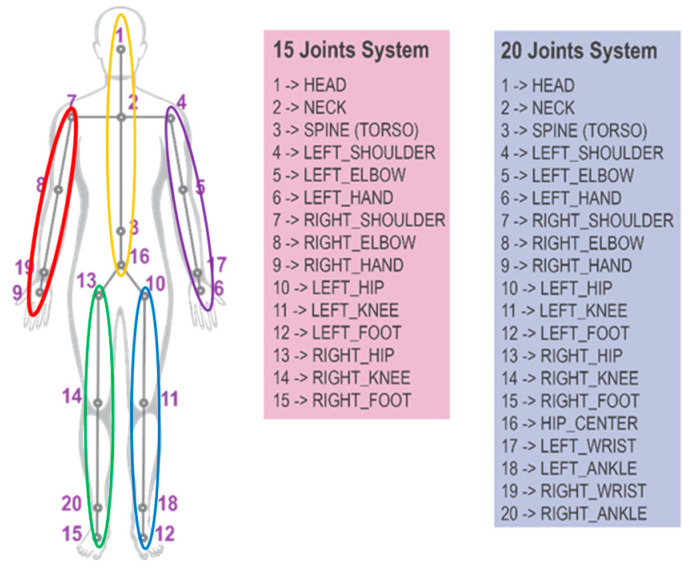
Five body parts of human structure. (Typical skeletal body joints models and the simplified 15/20 joints system).

In this section, we divided the whole human body into five main parts, considering the physical structure of the human body for mining more representative spatial–temporal features, as shown in Figure 5. Instead of taking the whole skeleton as the input of the deep learning model, we established five body parts, including left arm (LA), right arm (RA), left leg (LL), right leg (RL) and trunk.

(5) $$s_{t}^{\left(la\right)}=concat\left(\left[s_{t}^{8}-s_{t}^{7},s_{t}^{19}-s_{t}^{7},s_{t}^{9}-s_{t}^{7}\right]\right),t\in T$$

(6) $$s_{t}^{\left(ra\right)}=concat\left(\left[s_{t}^{5}-s_{t}^{4},s_{t}^{17}-s_{t}^{4},s_{t}^{6}-s_{t}^{4}\right]\right),\;t\in T$$

(7) $$s_{t}^{\left(ll\right)}=concat\left(\left[s_{t}^{14}-s_{t}^{13},s_{t}^{20}-s_{t}^{13},s_{t}^{15}-s_{t}^{13}\right]\right),t\in T$$

(8) $$s_{t}^{\left(rl\right)}=concat\left(\left[s_{t}^{11}-s_{t}^{10},s_{t}^{18}-s_{t}^{10},s_{t}^{12}-s_{t}^{10}\right]\right),t\in T$$

(9) $$s_{t}^{\left(t\right)}=concat\left(\left[s_{t}^{2}-s_{t}^{1},s_{t}^{3}-s_{t}^{1},s_{t}^{16}-s_{t}^{1}\right]\right),t\in T$$

(10) $$x_{t}^{\left(b\right)}=concat\left(s_{t}^{\left(la\right)},s_{t}^{\left(ra\right)},s_{t}^{\left(ll\right)},s_{t}^{\left(rl\right)},s_{t}^{\left(t\right)}\right)$$

#### 3.2.2. Joint-Level Input

**Figure 6:**
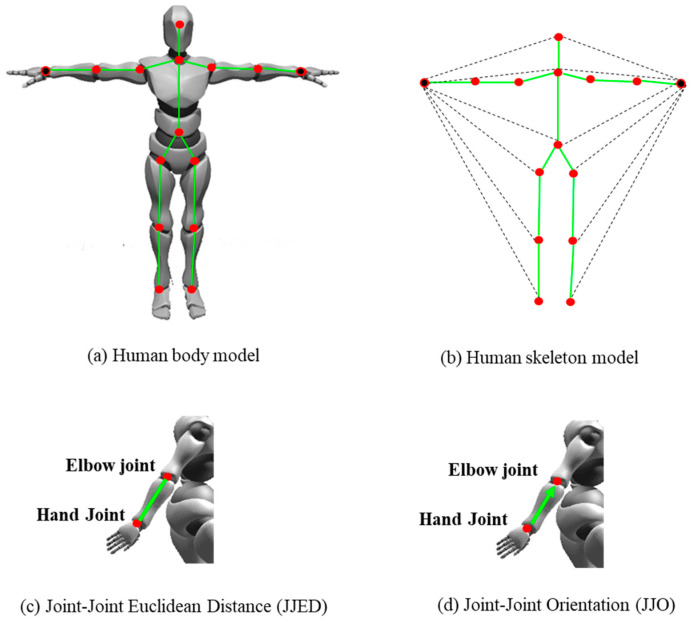
Schemes of distance and orientation.

Construction tasks are very heavily reliant on manual actions of workers, such as “installation” or “tying retar”. Therefore, the relationship between the two hands and the relationship between the hands and other joints are highly important to distinguish construction worker actions. Hence, we chose two representations of geometric features, Joint–Joint Euclidean Distance (JJED) and Joint–Joint Orientation (JJO), as the input of this stream. JJD represents the Euclidean distance between any joints within a 20-joint model with two hands, significantly reflecting the relative relationship between directly or indirectly connecting with the hands. JJO represents the x, y, and z orientations from any joints to the two hands within a 20-joint model, and the values are calculated with the unit length vector, as shown in Figure 6.

#### 3.2.3. Motion Data

**Figure 7:**
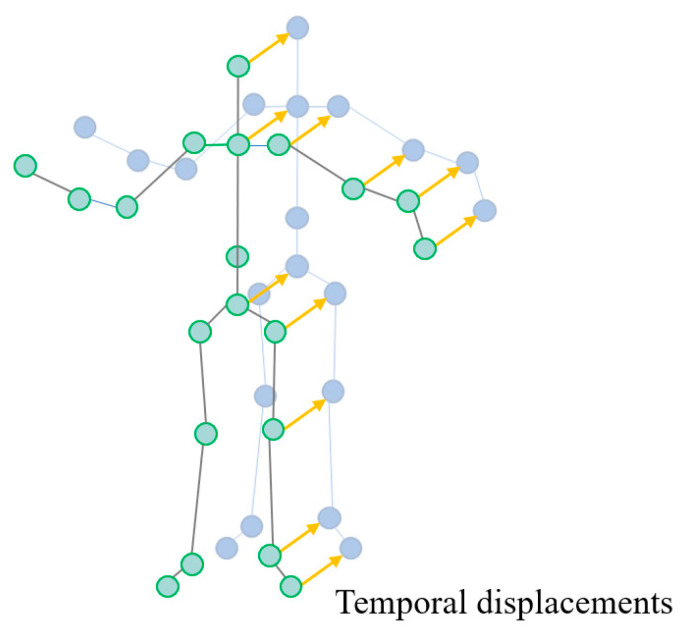
The motion data of skeleton with temporal displacements.

Specifically, the raw skeleton at frame t is represented as $P^{t}\in {\mathbb{R}}^{N\times 3}$, where N is the number of joints, $P_{i}^{t}\in {\mathbb{R}}^{3}$ denotes the 3D coordinates of i−th joint at time t. Then, the joint accelerations at time t can be calculated as $A^{t}=V^{t+1}-V^{t}$, where $V^{t}=P^{t+1}-P^{t}$. $V^{t}$ and $A^{t}$ can be considered as the first-order and second-order derivatives of the joint coordinates, respectively. Temporal interpolation is applied to the velocity and acceleration information to have a consistent sequence length, as shown in Figure 7.

## 4. Results and Discussion

### 4.1. Dataset and Implementation Details

To assess the effectiveness of our model, comprehensive experiments were carried out using the Construction Motion Library (CML) dataset [45], tailored for motion recognition in construction-related actions. The CML dataset comprises a vast collection of over 61,275 samples, equivalent to approximately 10 million frames, encompassing 73 distinct action classes performed by around 300 individual subjects. Each sample offers motion data represented by 20-joint skeletons. The dataset is broadly categorized into four fundamental activity types: production activities, unsafe activities, awkward activities, and common activities, as shown in Table 1.

The modeling was performed on a desktop computer equipped with an i7-11700@ 2.50 GHz CPU and a GeForce GTX 3060Ti GPU. The CML dataset comprised a total of 61,275 samples, which were randomly shuffled and divided into a training set (70%) and a testing set (30%). The training data were further segmented into a training subset (60%) and a validation subset (10%). To mitigate overfitting, parameter tuning involved adjusting values while monitoring optimization loss and accuracy on both the training and validation sets. The training employed the adaptive moment estimation (Adam) optimizer [46] to dynamically adjust the learning rate during the training process, with a weight decay set at 0.0005. A batch size of 256 and a learning rate of 0.00001 were set for the experiment. Additionally, several effective techniques, such as Batch Normalization [47], early stopping, and dropout with a probability of 0.5, were applied to prevent overfitting.

### 4.2. Performance of Multi-Stream Network

#### 4.2.1. Overall Performance of Different Inputs

**Figure 8:**
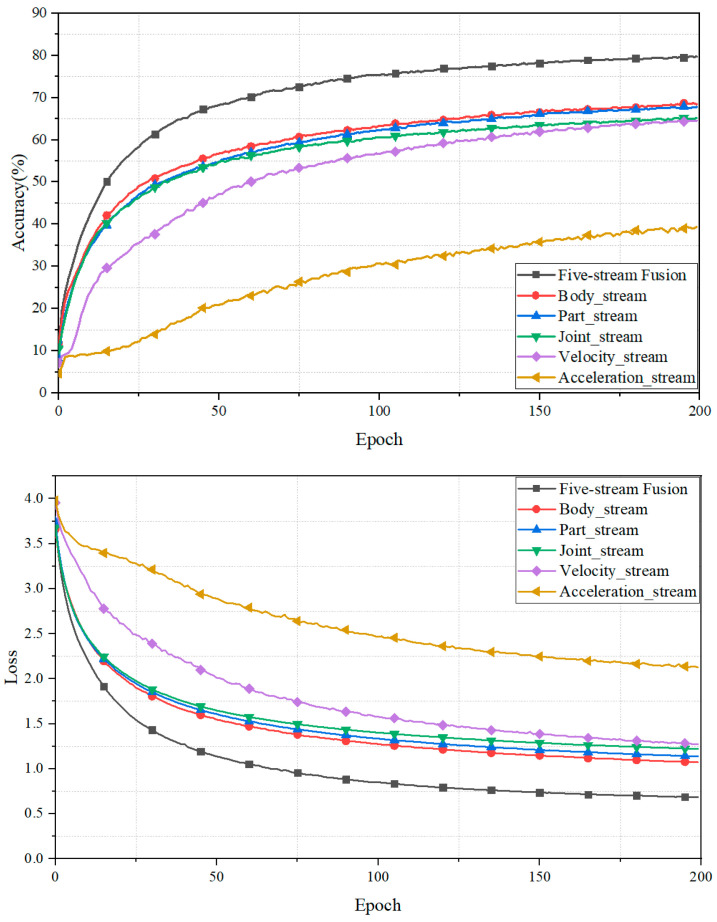
The validation set’s accuracy and loss over training epochs.

In this section, we evaluate the accuracy of various input streams, as described in Section 3.2. The results provide the following insights. Firstly, we observed that the five-stream fusion achieved the highest accuracy, suggesting that a greater number of input features allow the model to capture valuable local and global connections, as shown in Figure 8. Secondly, the motion data input exhibited suboptimal performance, especially for the acceleration input. This could be attributed to the model’s heightened focus on potential dependencies among the vertices of human joints in the temporal domain. Lastly, both the body input stream and the velocity input stream outperformed other input features within the same branch. More details about the input feature combinations are presented in Table 2. Furthermore, Table 3 gives an overview of each model’s average F1 score, precision, and recall focusing on different input streams. The average precision, recall, and F1 score of the 5-stream fusion input was around 79% which shows the efficacy of our proposed action recognition system.

In this research, we have introduced a novel two-stage feature fusion method aimed at enhancing the integration of multi-stream features, as depicted in Figure 1. This approach diverges from many existing studies that directly concatenate all input streams in the final stage. Specifically, in the early fusion stage, we combined temporal and spatial dimension features independently. Within the spatial domain, we amalgamated features at four distinct scales (body, part, joint), enabling the network to effectively explore spatial characteristics. In the temporal domain, our emphasis lay in motion information obtained by combining velocity and acceleration features. In the late fusion stage, we consolidated features from the early stage to investigate the correlation between these two feature types. The results illustrate that the two-stage fusion strategy outperforms direct concatenation, resulting in an improvement of approximately 1.25%, as shown in Table 4.

#### 4.2.2. Performance of Bottleneck Structure

In Section 3.1.2, we introduced the bottleneck structure into each TCN bottleneck to reduce the model size and computational cost. The bottleneck structure includes a hyper-parameter, the reduction rate (r), which determines the number of channels in the middle layers. To elaborate, considering the fact that the input and output channels are both 32 and choosing a channel reduction rate of r = 2, along with temporal window sizes of 3 and 5, the basic block in this research contains 8192 parameters, whereas the bottleneck block contains only 2304 parameters, or nearly half the parameters of the basic block. Further details are presented in Table 5.

We conducted comparative experiments to illustrate the influence of the bottleneck structure, as presented in Table 6. The results unmistakably indicate that the bottleneck structure, especially with a suitable reduction rate (r = 2/4), effectively reduces the model’s complexity, achieving a significant reduction—of half or even a quarter—in the model parameters while maintaining a reasonably tolerable decline in model accuracy. This outcome aligns with the findings in [48].

#### 4.2.3. Performance with and without Attention

**Figure 9:**
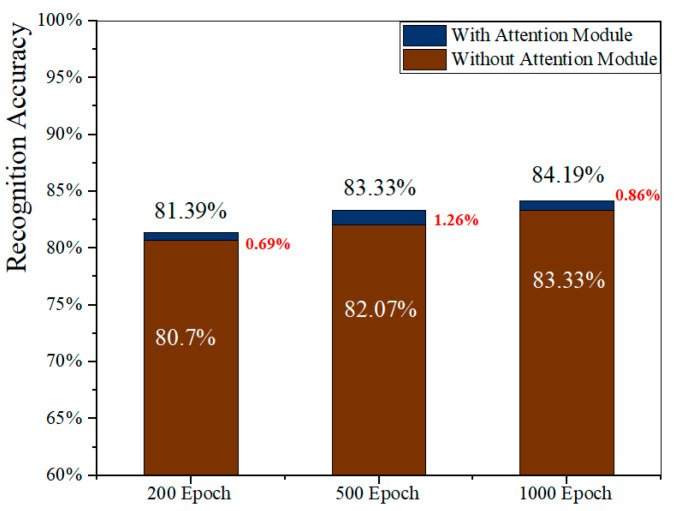
Comparison of accuracy with or without attention block.

In the discussion in Section 3.1.3, we emphasized that the attention mechanism serves as an information selection mechanism. By considering the varying degrees of importance among correlated features, the attention model assigns distinct weights to each input stream, enabling the model to adaptively focus on critical information. As depicted in Figure 1, three attention blocks were incorporated individually. To emphasize the advantages of the attention model, we conducted a comparison of various experiments, including the proposed stream attention block and benchmark experiments, as illustrated in Figure 9. The results distinctly demonstrate that the recognition accuracy exhibited the highest increase, approximately 1.26% after 500 epochs, further validating the necessity and efficacy of the attention module.

#### 4.2.4. Comparison with Other State-of-the-Art Action Recognition Models

In terms of broader impact, this research conducted a series of comparison experiments with existing classical action recognition methods. One notable method, ST-GCN [11], stands out, as it was the first to employ graphs for extracting dynamic information from human body skeletons, achieving high accuracy in benchmark datasets for skeleton-based deep learning. ST-GCN employs nine ST-GCN blocks for spatial and temporal graph convolutions, followed by fully connected dense layers and a SoftMax classifier for action prediction. In this study, the key modification lies in the input graphs, along with the introduction of multi-stream and attention mechanisms to enhance accuracy. Reproducing ST-GCN on the same CML skeleton dataset showed that our approach outperformed ST-GCN by 4.68%, affirming the effectiveness of our multi-stream model in recognizing construction workers’ actions. Meanwhile, to ensure a fair comparison, our method was also compared to four dynamic modeling models, including the traditional RNN [4] and two-layer LSTM [6], as shown in Table 7. The traditional RNN showed the poorest performance, with 72.71 accuracy, while various two-layer LSTMs exhibited better accuracy, though they still lagged behind our model by about 6.82%. This discrepancy may stem from the fact that these methods primarily focus on modeling the temporal dynamics of actions, without fully considering the spatial characteristics unique to construction workers.

## 5. Conclusions

In this study, we have presented a novel model with two key innovations. Firstly, we introduced a multi-scale graph strategy to capture local and contour features, effectively utilizing three levels of spatial features. Additionally, we incorporated two levels of motion data to comprehensively capture motion dynamics. Next, the proposed fused multi-stream architecture integrates five spatial–temporal feature sequences deriving from raw skeleton data, including body-level, part-level, joint-level, velocity, and acceleration features. Our experiments have demonstrated the effectiveness of employing additional input features, leading to notable improvements in recognition accuracy. We observed that intra-frame features (body-level input + part-level input + joint-level input) outperformed inter-frame features (velocity and acceleration) by about 10.41%. Moreover, both the body-level stream and velocity stream yielded superior results compared to the other individual features. The five-stream direct fusion strategy achieved the highest accuracy, at about 79.75%. Finally, we also added three attention modules to further improve the accuracy of the whole model, and this enabled our model to extract deep and rich features, significantly enhancing its action recognition performance.

In summary, the recognition of construction workers’ activities is of paramount importance in optimizing project task allocation and safeguarding the long-term health of the workforce. Delving into the impact of deep learning frameworks on skeleton-based automated recognition techniques is a fundamental step in ensuring the safety and productivity of human workers. With the proliferation of tools for extracting and processing human skeleton information in the construction sector, the potential and desirability of automated recognition techniques in future construction sites are evident. This research underscores the effectiveness of the multi-scale graph strategy and the fusion of diverse spatial–temporal features in enhancing action recognition models. These advancements offer the potential not only to refine action recognition but also to make substantial contributions to enhancing workplace safety and efficiency, particularly within the construction industry.

## Figures and Tables

**Table 1:** Statistics of the CML Dataset.

	Construction-Related Activities	Unsafe Activities	Awkward Activities	Production Activities	Common Activities
Number of labels	73	38	10	12	13
Number of samples	61,275	36,778	5101	5105	14,291
File size	10.53 GB	5.98 GB	0.69 GB	0.72 GB	3.14 GB

**Table 2:** Comparison of the action recognition accuracy of different geometric features.

Input Feature	200 Epoch (Acc%)	Input Feature	200 Epoch (Acc%)
Body + part + joint + velocity + acceleration	79.75	Body + velocity	74.29
Body	69.13	Body + velocity	72.63
Part	67.55	Part + velocity	75.07
Joint	64.86	Part + acceleration	71.18
Velocity	63.94	Joint + velocity	75.79
Acceleration	39.21	Joint + acceleration	69.87
Body + part + joint	76.02	Body + part + velocity	77.96
Velocity + acceleration	65.61	Body + part + acceleration	76.35
Body + part + joint + acceleration	78.66	Body + part + joint + velocity	79.01

**Table 3:** The recall, precision, and F1 scores of action recognition classifications.

Metrics	5-Stream Fusion	Body Stream	Part Stream	Joint Stream	Velocity Stream	Acceleration Stream
Recall	0.7875	0.6905	0.6558	0.6432	0.6485	0.3934
Precision	0.8012	0.7013	0.6695	0.6498	0.6278	0.3947
FI score	0.7760	0.6872	0.6743	0.6444	0.6300	0.3853

**Table 4:** The comparison of results of two-stage fusion strategy and directly concatenating.

Recognition Accuracy	Naive Concatenate	Two-Step Fusion Strategy	Difference
Acc (%)	79.45	80.7	1.25

**Table 5:** Comparison with different reduction rates (r) and parameter numbers.

Reduction Rate (r)	Parameter	Total Param.	Basic Param.	Ratio
r = 2	K = 3, 32 * 16 * 1 + 16 * 16 * 5 + 32 * 16 * 1 = 2304	4096	8192	0.5
	K = 5, 32 * 16 * 1 + 16 * 16 * 3 + 32 * 16 * 1 = 1792			
r = 4	K = 3, 32 * 8 * 1 + 8 * 8 * 3 + 32 * 8 * 1 = 704	1536	8192	0.1875
	K = 5, 32 * 8 * 1 + 8 * 8 * 5 + 32 * 8 * 1 = 832			
r = 8	K = 3, 32 * 4 * 1 + 4 * 4 * 3 + 32 * 4 * 1 = 304	640	8192	0.078125
	K = 5, 32 * 4 * 1 + 4 * 4 * 5 + 32 * 4 * 1 = 336			

**Note**: * represents multiplication operation.

**Table 6:** Illustration of the influence of the bottleneck structure.

	No Bottle	R = 2	R = 4	R = 8
Acc (%)	80.7	79.92	78.79	76.82

**Table 7:** Illustrates the influence of the bottleneck structure.

Algorithms	Acc (%)
ST-GCN	79.51
2-layer LSTM	77.37
Traditional RNN	72.71
Our approach	84.19

## Data Availability

All data generated or analyzed during this study are included in this published article.
